# Risk factors associated with progressive increases in serum creatinine concentrations in cats with cancer receiving doxorubicin

**DOI:** 10.1111/jvim.15867

**Published:** 2020-08-11

**Authors:** Lucy Kopecny, Carrie A. Palm, Katherine A. Skorupski, Mikel Delgado, Robert B. Rebhun

**Affiliations:** ^1^ Department of Veterinary Medicine and Epidemiology University of California, Davis Davis California USA; ^2^ Department of Veterinary Surgical and Radiological Sciences University of California, Davis Davis California USA

**Keywords:** chemotherapy, chronic kidney disease, feline, nephrotoxicosis

## Abstract

**Background:**

Azotemia occurs in cats administered doxorubicin, but risk factors have not been explored.

**Objective:**

To determine incidence of progressive increases in serum creatinine concentration in cats with cancer receiving doxorubicin in single or multiagent chemotherapy protocols and associated risk factors.

**Animals:**

Seventy cats with cancer receiving doxorubicin.

**Methods:**

A retrospective study (2007‐2017) of cats with indices of kidney function recorded before and after doxorubicin administration was reviewed. Cats diagnosed with kidney injury because of known etiologies other than possible doxorubicin toxicosis were excluded. Variables were compared to identify risk factors.

**Results:**

Mean age (±SD) was 10.9 years (±3.2). Cancer types included lymphoma (n = 36), sarcoma (n = 19) and carcinoma (n = 14). Chronic kidney disease was present in 29/70 (41%) cats before receiving doxorubicin. Of 70 cats, 24 (34%) developed an increase in serum creatinine concentration ≥0.3 mg/dL and 10 (14%) had an increase ≥50% from baseline. Mean time to increases in serum creatinine concentration ≥0.3 mg/dL from first administration of doxorubicin was 119.3 days (±89.7), with mean 2.8 (±1.2) doses administered. Neutropenia or anemia during chemotherapy and number of radiation therapy treatments under general anesthesia were risk factors for increases in serum creatinine concentration (*P* < .05). Cats receiving single agent doxorubicin had a higher likelihood of an increase in serum creatinine concentration ≥0.3 mg/dL from baseline than cats receiving CHOP‐based chemotherapy protocols (OR 20.0, 95% CI 2.9‐100).

**Conclusions and Clinical Importance:**

Progressive increases in serum creatinine concentration from baseline were common in cats receiving doxorubicin and associated risk factors were identified.

**Abbreviations**
AKI: acute kidney injury
BCS: body condition score
CI: confidence interval
CKD: chronic kidney disease
FeLV: feline leukemia virus
FIV: feline immunodeficiency virus
IRIS: International Renal Interest Society
NSAID: nonsteroidal anti‐inflammatory drug
OR: odds ratio
SDMA: symmetric dimethylarginine
UC Davis VMTH: University of California, Davis William R. Pritchard Veterinary Medical Teaching Hospital
VCOG‐CTCAE: Veterinary Cooperative Oncology Group Common Terminology Criteria for Adverse Events v1.1

## 1 INTRODUCTION

Doxorubicin is an anthracycline antibiotic that is used in both single agent and multiagent chemotherapeutic protocols for treatment of a variety of cancers in cats.^1^ Doxorubicin‐associated nephrotoxicosis, as defined by development of overt azotemia, occurs in cats, rabbits and pigs.^2^, ^3^, ^4^, ^5^ In an experimental study of healthy, young adult cats administered doxorubicin at a dose of 30 mg/m^2^ every 21 days to a cumulative dose of 300 mg/m^2^, serum creatinine concentrations increased outside the reference interval in 2/6 cats and serum creatinine clearance decreased significantly in all cats during the study period. For the 2 cats that became azotemic, serum creatinine concentration increased from 1.0 and 1.1 mg/dL to 1.6 and 2.6 mg/dL, respectively. These cats had mild to moderate mesangial matrix thickening, tubular cell necrosis and tubular regeneration, with some cats also having mild to moderate nonsuppurative interstitial nephritis.^2^ Development of azotemia occurs in cats receiving doxorubicin for treatment of various cancers,^6^ with suggestions that this is in part associated with the cumulative dose.^6^, ^7^ In cats with vaccine‐associated sarcoma, 5/55 (9%) cats became azotemic a median of 8 months after beginning single agent doxorubicin chemotherapy.^6^ Two of 17 (12%) of cats with several different cancer types became azotemic after receiving >100 mg/m^2^ cumulative doxorubicin dose (combined with cyclophosphamide).^7^ Ten of 60 (17%) cats receiving doxorubicin based on either bodyweight or body surface area became azotemic, though there was no difference with regard to BUN and serum creatinine concentrations between groups.^9^

In humans, doxorubicin is not associated with nephrotoxicosis, however, other chemotherapeutic agents, including cisplatin, mitomycin C, gemcitabine and ifosfamide, can be nephrotoxic.^9^ Several patient‐related factors increase the risk and severity of drug‐induced kidney injury. Risk factors include age and female sex as both are associated with decreased muscle mass and total body water and therefore likely an underrepresentation of pre‐existing kidney dysfunction, as defined by serum creatinine concentration, before administration of chemotherapy. Intravascular volume depletion from vomiting, diarrhea and diuretics also increases the risk of drug‐induced nephrotoxicosis. Cancers such as multiple myeloma, lymphoma and leukemia and diabetes mellitus are associated with increased risk of nephrotoxicosis.^9^

While doxorubicin‐induced nephrotoxicosis occurs in cats, risk factors for its development have not been thoroughly evaluated. In addition, previous studies have defined decreased kidney function by serum creatinine concentration outside of laboratory reference intervals, which identifies disease late in its course after considerable injury and function loss have occurred. The aims of this retrospective study were to determine the incidence of early loss of kidney function (as defined by ≥0.3 mg/dL increase in serum creatinine concentration) in cats with cancer receiving doxorubicin in single or multiagent chemotherapy protocols and to determine risk factors associated with these progressive elevations in serum creatinine concentration. We hypothesized that pre‐existing chronic kidney disease, radiation therapy, recent anesthesia or surgery and cumulative dose of doxorubicin would be risk factors for increasing serum creatinine concentrations in cats receiving doxorubicin.

## 2 MATERIALS AND METHODS

Medical records of cats that received 1 or more doses of doxorubicin at the William R. Pritchard Veterinary Medical Teaching Hospital, University of California, Davis (UC Davis VMTH) from 2007 to 2017 were reviewed. Cats were excluded if serum creatinine concentration was not measured before or after doxorubicin administration or if serum creatinine concentration was not measured at the UC Davis VMTH. Cats with known or suspected renal lymphoma were also excluded. Renal lymphoma was defined as any cat with cytologic or necropsy confirmation of renal lymphoma or cats where serum creatinine concentration improved with initiation of chemotherapy and where serum creatinine concentration worsened when lymphoma relapsed. Cats were additionally excluded if a cause of kidney injury (eg, ureteral obstruction) other than possible doxorubicin toxicosis was identified or if serum creatinine concentration increased by ≥0.3 mg/dL from baseline before any administration of doxorubicin. Cats were monitored for up to 6 months after completion of the chemotherapy protocol. Indices of kidney function (including serum creatinine and BUN concentrations) were assessed during and after chemotherapy protocols at the discretion of the attending clinician. A sustained increase in serum creatinine concentration by ≥0.3 mg/dL from baseline at any time point after 1 or more doses of doxorubicin was considered to indicate kidney injury, as defined by the International Renal Interest Society (IRIS) acute kidney injury (AKI) guidelines.^10^ This value was selected to increase detection of decreased renal function, particularly since many cats with cancer experience weight loss, muscle mass loss or both^11^ and muscle mass is recognized to impact serum creatinine concentration. In order to be defined as a sustained increase, serum creatinine concentration had to be increased on more than 1 sequential measurement by ≥0.3 mg/dL from baseline. This was chosen to exclude cats with transient increases such as from prerenal causes. Acute kidney injury was also assessed using the Veterinary Cooperative Oncology Group Common Terminology Criteria for Adverse Events (VCOG‐CTCAE) v1.1.^12^ The time at which a sustained increase in serum creatinine concentration ≥0.3 mg/dL from start of chemotherapy was recorded. Staging of chronic kidney disease (CKD) was based on IRIS guidelines^13^ using clinicopathologic and, where available, ultrasonographic data; symmetric dimethylarginine (SDMA) was not used for staging in this cohort of cats as it was not regularly available. Specifically, cats were diagnosed with IRIS stage 1 CKD where serum creatinine concentration was <1.6 mg/dL, but there were ultrasonographic changes consistent with CKD such as decreased kidney size, irregular contour, increased echogenicity or decreased corticomedullary differentiation, with or without decreased urine concentration (urine specific gravity <1.035).^13^, ^14^

Signalment (including breed, sex, neuter status and age at initiation of chemotherapy) was recorded. Where available, feline immunodeficiency virus (FIV) antibody and feline leukemia virus (FeLV) antigen status were noted. Comorbid disorders known to be risk factors for AKI in humans undergoing chemotherapy or cats were recorded, including diabetes mellitus, urinary tract infection and hyperthyroidism.^9^, ^15^, ^16^, ^17^ Data collected from each visit included clinical values (bodyweight, appetite, presence of vomiting or diarrhea, hydration status, body condition score [BCS]), clinicopathologic data (hematocrit, neutrophil count and serum creatinine concentration), doxorubicin dose and any concurrent administration of nephrotoxic drugs (eg, nonsteroidal anti‐inflammatory drugs [NSAIDs] and angiotensin converting enzyme inhibitors). Changes in bodyweight over the course of the study were evaluated for each cat. Muscle condition score was not available.

Continuous variables were assessed for normality using the Shapiro‐Wilk test and visual inspection of Q‐Q plots. If normally distributed, mean (±SD) values and if not normally distributed, median (range) values were used to describe these variables. *T* tests were used to compare the cumulative doxorubicin dose administered in mg/m^2^ for cats that had an increase in serum creatinine concentration ≥0.3 mg/dL from baseline and cats that had <0.3 mg/dL increase in serum creatinine concentration from baseline during chemotherapy.

Variables considered to be possible risk factors for an increase in serum creatinine concentration ≥0.3 mg/dL from baseline were evaluated using a logistic regression model. The evaluated variables included age, bodyweight, baseline serum creatinine concentration, IRIS stage of CKD (at the time of initial evaluation for all listed variables); presence of metastatic disease; anesthesia for surgery, advanced imaging or an endoscopic procedure within 30 days of starting chemotherapy; radiation therapy and number of radiation therapy treatments within 30 days of starting chemotherapy; chemotherapy protocol and anemia (hematocrit <30%) or neutropenia (neutrophil count <2000/μL) occurring during chemotherapy. A backward elimination procedure was used. Nonstatistically significant variables with the highest *P*‐value were eliminated at each step until all predictors in the model had *P*‐values <.05.

ANOVA was used to assess the relation between different chemotherapeutic protocols and time to increase in serum creatinine concentration ≥0.3 mg/dL from baseline. *T* tests were used to compare age, mean number of doses of doxorubicin, mean inclusion times in the study and mean survival times for cats receiving doxorubicin only to cats that received CHOP.

Statistical analyses were conducted using SAS University Edition (SAS Institute Inc., Cary, North Carolina). *P*‐values <.05 were considered significant.

## 3 RESULTS

**FIGURE 1:**
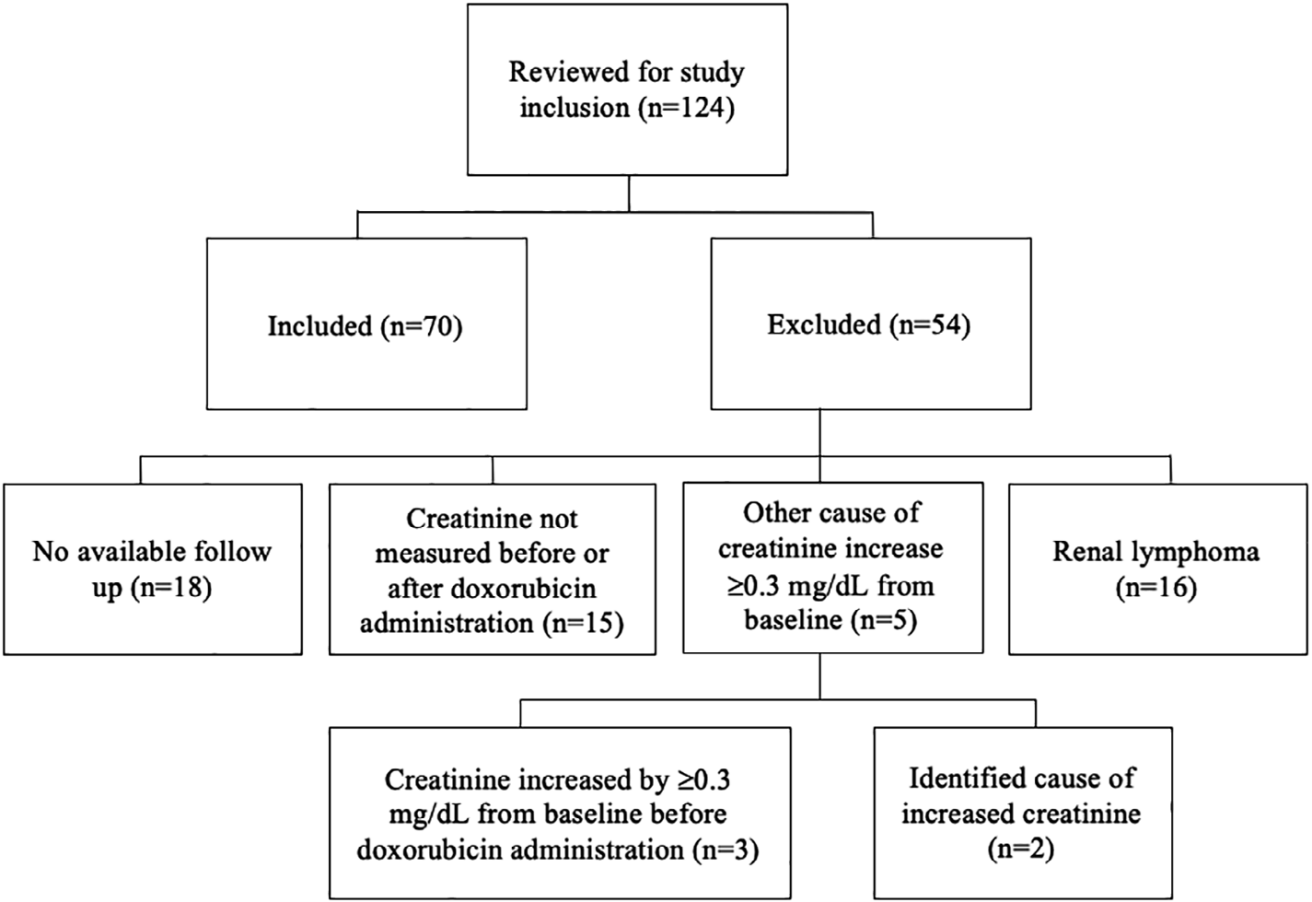
Summary of included and excluded cats with cancer receiving doxorubicin in single or multiagent chemotherapy protocols

Medical records of 124 cats were reviewed for inclusion. A total of 70 cats met the inclusion criteria. Fifty‐four cats were excluded because of known or suspected renal lymphoma (n = 16), no available follow‐up (n = 18), indices of kidney function not being measured before or after doxorubicin administration (n = 15) or confirmed noncancer related causes of kidney injury such as ureteral obstruction (n = 2) (Figure 1). Three additional cats were excluded that had increases in serum creatinine concentration ≥0.3 mg/dL from baseline before receiving doxorubicin, 2 of which were receiving CHOP‐based chemotherapy protocols.

Mean age (±SD) of included cats was 10.9 years (±3.2, range, 0.9‐17.1). Of 70 cats, 39 (56%) were female spayed, 30 (43%) male castrated and 1 (1%) male intact. Most common breeds were domestic shorthair (47/70, 67%), domestic longhair (10/70, 14%) and domestic medium hair (7/70, 10%). Other breeds represented were Siamese or Siamese‐cross (4/70, 6%), Abyssinian (1/70, 1%) and Manx (1/70, 1%). Median initial weight was 4.4 kg (range, 2.2‐9.1). Median weight change was −0.4 kg (range, −2.9‐0.6), with median final weight at last follow‐up 4.0 kg (range, 2.1‐9.1). Of 70 cats, initial BCS was available in 61, with median initial BCS 5/9 (range, 2‐9). At follow‐up, BCS was available in 62/70 cats with median BCS 4.5/9 (range, 2‐8). Median time from inclusion to last follow‐up was 123 days (range, 21‐477), with a total of 383 visits included. The mean number of times serum creatinine concentration was measured in each cat was 5.2 (±1.8).

Testing for FIV antibodies and FeLV antigens was performed in 33/70 and 30/70 cats, respectively. Of 33 cats tested for FIV antibodies, 4 (12%) were positive and of 30 cats tested for FeLV antigens, 1 (3%) was positive. Two cats had diabetes mellitus. Three cats were treated for hyperthyroidism with methimazole while receiving chemotherapy; 2 were diagnosed before starting chemotherapy and 1 was diagnosed with hyperthyroidism during treatment. The cat diagnosed with hyperthyroidism during the chemotherapy protocol had stable serum creatinine concentration when total T4 concentration was mildly decreased (0.9 μg/dL, reference range, 1.1‐3.9). This cat developed progressive increases in serum creatinine concentration from baseline 209 days after starting methimazole. Two cats had a positive urine culture at the time their chemotherapy protocol was initiated; both were treated with antimicrobials based on urine culture results with follow‐up negative urine culture results. One cat had a positive urine culture during the chemotherapy protocol; this cat did not experience any increase in serum creatinine concentration during the study period.

Appetite was decreased in 51/380 (13%) instances where it was recorded. Vomiting was recorded as occurring since the previous visit in 61/376 (16%) instances and diarrhea in 25/376 (7%) instances. Hydration status at the time of each included visit was assessed in 380 instances and in 21 of these instances (6%), the cat was assessed to be at least 5% dehydrated. Only 4/21 instances of a cat being dehydrated coincided with that cat developing an increase in serum creatinine concentration ≥0.3 mg/dL from baseline at some point.

Types of cancer and chemotherapy protocol used for treatment are provided in Table 1. Of 70 cats, 24 cats (34%) were neutropenic at least once during their chemotherapy protocol. Thirty‐one cats (44%) were anemic with hematocrit <30% during their chemotherapy protocol, with 11 of these cats having a hematocrit <25%.

**TABLE 1:** Types of cancer diagnosed and chemotherapy protocol used for cats included in the study (n = 70)

		Number (%)
Cancer type	Lymphoma	36 (51)
	Injection site sarcoma	15 (21)
	Mammary carcinoma	7 (10)
	Small or large bowel adenocarcinoma	6 (9)
	Hemangiosarcoma	3 (4)
	Cutaneous squamous cell carcinoma	1 (1)
	Rhabdomyosarcoma	1 (1)
	Peripheral nerve sheath tumor	1 (1)
Chemotherapy protocol type	CHOP with or without l‐asparaginase	36 (51)
	Single agent doxorubicin	28 (40)
	Doxorubicin and carboplatin	6 (9)

Of 70 cats, 41 (59%) underwent anesthesia within 30 days of starting chemotherapy (not including cats anesthetized for radiation therapy). These anesthetic events were for surgery (n = 37), advanced imaging (magnetic resonance imaging with contrast administration or computed tomography with contrast administration, n = 8), esophagostomy tube placement (n = 1) or 1 or more of these. Of these 41 cats undergoing anesthesia, 11 then underwent radiation therapy under general anesthesia. Radiation therapy under general anesthesia was performed in 12 cats (17%) before (n = 3) or during the chemotherapy protocol (n = 9), with cats undergoing median 15 radiation treatments under general anesthesia (range, 4‐20). In total, 18/41 (44%) cats undergoing general anesthesia (not for radiation therapy) within 30 days of starting chemotherapy and 8/12 (67%) cats undergoing radiation therapy under general anesthesia had an increase in serum creatinine concentration ≥0.3 mg/dL from baseline.

Before starting chemotherapy, 2 cats were receiving NSAIDs, which were discontinued at the time chemotherapy was initiated. Neither cat had an increase in serum creatinine concentration ≥0.3 mg/dL from baseline. One cat received meloxicam while receiving a combination of doxorubicin and carboplatin chemotherapy for recurrent fibrosarcoma; this cat's serum creatinine concentration increased from 1.1 to 5.5 mg/dL. No other known nephrotoxic drugs were administered to cats in this study while they were receiving doxorubicin.

Based on IRIS staging of CKD, 23/70 (33%) cats had stage 1 CKD and 6/70 (9%) cats had stage 2 CKD before starting chemotherapy. No cat had stage 3 or 4 CKD because doxorubicin is typically withheld in cats with pre‐existing azotemic CKD at our institution. Before administration of doxorubicin, 41/70 (59%) cats had no evidence of CKD using IRIS staging guidelines. Median initial serum creatinine concentration was 1.2 mg/dL (range, 0.6‐2.2; reference range, 1.1‐2.2 mg/dL). Median serum creatinine concentration at final follow‐up was 1.5 mg/dL (range, 0.7‐5.5), with median change in serum creatinine concentration from baseline 0.1 mg/dL (range, −0.6‐4.4). Of 70 cats, 24 (34%) had a sustained increase in serum creatinine concentration ≥0.3 mg/dL and 10 cats (14%) had a sustained increase in serum creatinine concentration ≥50% from baseline. Of 24 cats with an increase in serum creatinine concentration ≥0.3 mg/dL from baseline, the median increase in serum creatinine concentration from baseline was 0.7 mg/dL (range, 0.3‐4.4) and 18 cats (26%) had serum creatinine concentration ≥1.6 mg/dL at last follow‐up (median 2.5 mg/dL, range, 1.6‐5.5). Grading of acute kidney injury using VCOG‐CTAE is presented in Table 2. Using this grading system, overall, 20/70 cats (29%) developed an acute kidney injury while receiving doxorubicin. No cat experienced grade 4 or 5 toxicosis. There was no significant difference in bodyweight of cats that experienced an increase in serum creatinine concentration ≥0.3 mg/dL from baseline than cats that did not (*P* = .35).

**TABLE 2:** Veterinary Cooperative Oncology Group Common Terminology Criteria for Adverse Events (VCOG‐CTCAE) acute kidney injury grade for included cats (n = 70)

VCOG‐CTCAE acute kidney injury grade	Number (%)
No acute kidney injury	50 (71%)
Grade 1	11 (16%)
Grade 2	6 (9%)
Grade 3	3 (4%)

*Notes:* No cats had grade 4 or 5 acute kidney injury.

The mean number of doxorubicin doses that cats received was 3.1 (±1.4, range 1‐7). Mean time to an increase in serum creatinine concentration ≥0.3 mg/dL from baseline from the first dose of doxorubicin was 119.3 days (±89.7), with mean number of doses 2.8 (±1.2). The mean cumulative dose of doxorubicin per body surface area administered to cats that developed an increase in serum creatinine concentration ≥0.3 mg/dL from baseline was 55.9 mg/m^2^ (±26.0). When compared to the total cumulative dose for cats that had <0.3 mg/dL increase in serum creatinine concentration from baseline (mean 52.8 mg/m^2^ ± 29.0), there was no significant difference (*P* = .7). However, cats with an increase in serum creatinine concentration ≥0.3 mg/dL from baseline were evaluated for a mean of 207 days (±123.0), which was significantly longer than cats that did not experience any increase in serum creatinine concentration from baseline (mean 125.6 days ±83.0; *P* = .006). In 7 cats, doxorubicin administration was discontinued because of progressive increases in serum creatinine concentration above the reference interval (>2.2 mg/dL); median serum creatinine concentration increase from baseline for these cats was 1.2 mg/dL (range, 0.4‐4.4).

Independent variables associated with an increase in serum creatinine concentration ≥0.3 mg/dL from baseline as the dependent variable included in the logistic regression model were initial presenting age, weight, baseline serum creatinine concentration, IRIS CKD stage, presence of metastatic disease and anesthesia for surgery, advanced imaging or an endoscopic procedure within 30 days of starting chemotherapy, radiation therapy and number of radiation therapy treatments within 30 days of starting chemotherapy, chemotherapy protocol and anemia (hematocrit <30%) or neutropenia (neutrophil count <2000/μL) occurring during chemotherapy. The final model indicated that neutropenia (odds ratio [OR] 12.4, 95% confidence interval [CI] 2.0‐76.6), anemia (OR 4.2, 95% CI 1.1‐16.2) and the number of radiation treatments (OR 1.2, 95% CI 1.1‐1.4) were all related to increased likelihood of an increase in serum creatinine concentration ≥0.3 mg/dL from baseline.

Type of chemotherapy protocol also had a statistically significant effect, with cats receiving single agent doxorubicin having a higher likelihood of an increase in serum creatinine concentration ≥0.3 mg/dL from baseline than cats receiving CHOP‐based chemotherapy protocols (OR 20.0, 95% CI 2.9‐100). There was no significant difference in the time that cats receiving CHOP‐based chemotherapy protocols (mean 162.3 days ±113) were evaluated in the study period than cats that were receiving doxorubicin alone (mean 142.6 days ±103.9; *P* = .5). There was also no significant difference in survival time for cats that died during the study period that were receiving CHOP‐based chemotherapy protocols (mean 148.5 days ±108.9) compared to those receiving single agent doxorubicin (mean 157.1 days ±112.0; *P* = .8). The mean age of cats receiving CHOP‐based chemotherapy protocols was 10.7 years (±3.1) and the mean age of cats receiving single agent doxorubicin was 10.6 years (±3.3); this was not significantly different (*P* = .9). The mean number of doses of doxorubicin that cats undergoing CHOP‐based chemotherapy protocols received was 2.6 (±1.4), whereas this was 3.8 (±1.0) doses for cats receiving single‐agent doxorubicin chemotherapy (*P* = .0003).

Of 70 cats, 19 (27%) were alive at the end of the 6 month follow‐up period, 47 (67%) had either died or were euthanized and 6 (8.6%) likely died or were euthanized because of a marked decline in their clinical condition and poor quality of life at the last recorded visit at UC Davis VMTH. In 5/70 cats (7%), the attending clinician reported progressive renal disease as contributing to their death; this diagnosis was based on hyporexia, vomiting and weight loss not attributable to their neoplasia or other comorbidities. All of these cats had an increase in serum creatinine concentration ≥50% from baseline and in 3 of these cats, doxorubicin had been discontinued.

## 4 DISCUSSION

Increases in serum creatinine concentration from baseline occur commonly in cats receiving doxorubicin as part of a chemotherapy protocol for a variety of cancers. In the cohort of this report, 34% of cats had an increase in serum creatinine concentration of ≥0.3 mg/dL from baseline after a mean of approximately 119 days and 14% of cats had an increase in serum creatinine concentration of ≥50%. In total, 26% of cats had serum creatinine concentration increase to ≥1.6 mg/dL while receiving doxorubicin. Progressive kidney disease was reported as contributing to the death of 7% of cats in this study.

Between 9% and 17% cats receiving doxorubicin become azotemic based on traditional evaluation of serum creatinine concentration outside of its reference interval, without consideration of muscle loss.^6^, ^7^, ^8^ The number of cats reported in the present study experiencing increases in serum creatinine concentration both above their baseline (34%) and above 1.6 mg/dL (26%) is higher than those reported previously; this partly reflects that increases in serum creatinine concentration within the reference interval were considered, rather than solely development of azotemia. Serum creatinine concentration is not a sensitive indicator of kidney injury or early decreased renal function as it does not increase beyond the reference interval until approximately 75% of total renal mass is lost. However, as utilized in this study, individualizing evaluation by using each cat's serum creatinine concentration as a baseline and then trending serum creatinine concentration over time improves sensitivity. In dogs, use of serum creatinine concentration trending has allowed detection of median 27% decrease in glomerular filtration rate, as compared to an estimated 75% loss before overt azotemia outside of reference ranges develops.^18^ In addition, in human patients with pre‐existing CKD who experience AKI, use of absolute increases in serum creatinine concentration over baseline can allow for early AKI diagnosis.^19^ This is relevant in populations of middle‐aged to older cats, where CKD occurs commonly^20^ and emphasized in the present study where 41% of cats had IRIS stage 1 and early stage 2 CKD. An absolute increase in serum creatinine concentration above baseline was therefore selected to improve detection of cats with kidney injury and subsequent decreased glomerular filtration rate. In addition, considering that many cats with cancer experience weight loss, muscle mass loss or both^11^ (resulting in serum creatinine concentration overestimating renal function), use of serum creatinine concentration increasing outside of the reference interval for diagnosis of kidney disease becomes even more inaccurate. Indeed, cats in this report lost a median of 0.4 kg over the course of the study, making it likely that serum creatinine concentration underrepresented the degree of kidney injury and function loss that occurred in many included cats. It would have been ideal to measure SDMA given that this filtration marker is more sensitive and less affected by loss of muscle mass^21^; however, because of the retrospective nature of this study, SDMA values were not available.

Overall, VCOG‐CTCAE grading identified a similar proportion of cats developing acute kidney injury (29%) to the system based on IRIS AKI grading used in this study because of its definition of AKI including increases in serum creatinine concentration >0.3 mg/dL above baseline.

Several risk factors for increases in serum creatinine concentration from baseline in cats receiving doxorubicin were identified. Neutropenia as a risk factor for increases in serum creatinine concentration from baseline might reflect higher peak doxorubicin doses in affected cats, possibly because of individual differences in metabolism or pharmacokinetics, than in those that did not experience chemotherapy‐induced neutropenia. Chemotherapy‐induced neutropenia has been correlated with improved outcomes in human patients as well as in dogs, likely because of greater biologic effect in an individual patient for treatment of their cancer. A recent study of dogs receiving conventional doses of CHOP for lymphoma showed that dogs with chemotherapy‐induced neutropenia had significantly prolonged duration of remission and survival times.^22^, ^23^ Greater biologic effect in cancer‐killing in an individual patient might be translated to greater potential for nephrotoxicosis, as this end‐organ may be exposed to higher or more prolonged concentrations of drug. Anemia might be a risk factor for increases in serum creatinine concentration from baseline because of hypoxic injury to the kidney, particularly when combined with nephrotoxic drug administration, though could also reflect more severe concurrent disease.

Both older age and the presence of CKD increase the risk of drug‐induced AKI in humans.^9^ This was not apparent in the present feline population. Case selection bias might have affected the impact of CKD on doxorubicin‐associated nephrotoxicosis as cats with IRIS stage 3 and 4 CKD are generally not prescribed doxorubicin at our institution because of concern for nephrotoxicity. The lack of cats with IRIS stage 3 or 4 CKD could have resulted in underestimation of the risk of doxorubicin‐associated nephrotoxicosis in the study population. However, it is also possible that cats with IRIS stage 3 or 4 CKD do not have higher risk of nephrotoxicosis compared to other cats, meaning that doxorubicin is being unnecessarily withheld.

Both the number of radiation therapy treatments and the type of chemotherapy protocol affected the risk of increases in serum creatinine concentration from baseline in cats in this study. The number of radiation therapy treatments was linked to a small increased risk for serum creatinine concentration increases from baseline, possibly because of the number of anesthetic events, as general anesthesia can lead to ischemia‐associated kidney injury.^24^ Most of these cats had already undergone anesthesia for other procedures, which could have further increased the risk of progressive increases in serum creatinine concentration. Cats receiving doxorubicin alone had a higher likelihood of an increase in serum creatinine concentration ≥0.3 mg/dL from baseline than cats receiving CHOP‐based chemotherapy protocols. While it was initially hypothesized that this could be associated with shorter survival times of cats with lymphoma receiving CHOP protocols as compared to cats receiving doxorubicin alone (typically for treatment of sarcoma or carcinoma), there was no difference in survival times between these groups. The cats receiving CHOP‐based chemotherapy protocols, however, did receive significantly fewer doses of doxorubicin (and thus a lower cumulative dose) compared to cats receiving single agent doxorubicin and this difference might therefore reflect a dose dependent effect. Lower likelihood of increases in serum creatinine concentration in cats receiving CHOP‐based chemotherapy could also relate to the decreased dose intensity in contrast to protocols using single agent doxorubicin, where doxorubicin is administered every 21 days. Increased dose intensity in the latter protocols might not allow adequate time for recovery of injury to the kidney before administration of the next dose.

Cats with an increase in serum creatinine concentration ≥0.3 mg/dL from baseline were evaluated for a significantly longer time period than cats without an increase in serum creatinine concentration from baseline. This suggests that there might be a time dependent effect not just related to cumulative dose (cumulative dose did not differ between these groups) and that additional cats might have developed progressive increases in serum creatinine concentration from baseline if longer follow‐up periods were available.

In healthy cats, experimental doxorubicin‐associated nephrotoxicosis has been demonstrated based on changes to creatinine clearance as well as renal histopathologic abnormalities.^2^ Providing some support for these findings, development of azotemia is associated with doxorubicin administration in cats receiving this drug as part of chemotherapy protocols for cancer.^6^, ^7^ Progressive increases in serum creatinine concentration in cats in the present study were attributed to doxorubicin‐associated nephrotoxicosis, although similar to other clinical studies, this cannot be proven conclusively. Diagnostics, including urinalysis, urine culture and abdominal ultrasonography, were sometimes performed at the time of cancer diagnosis, but inconsistently when serum creatinine concentration either increased within or above the reference interval, suggesting that other causes of kidney injury, for example, pyelonephritis or ureteral obstruction, cannot be excluded in all cases. While cats with suspected or confirmed renal lymphoma were eliminated using strict exclusion criteria, it remains possible some cats with renal involvement of lymphoma might have been included in the study. The incidence of progressive increases in serum creatinine concentration in cats with cancer, particularly those undergoing chemotherapy protocols not including doxorubicin, is unknown.

Other limitations of the present study include its retrospective nature and small study population. It is recognized that use of an absolute increase in serum creatinine concentration of ≥0.3 mg/dL from baseline might have resulted in cats being falsely classified as having decreased glomerular filtration rate from baseline since serum creatinine concentrations can have normal daily variability. It could additionally vary because of mild dehydration unable to be detected on general physical examination. Serum creatinine concentration has been reported to vary in fed and fasted states as well as diurnally, and highest median within‐day variations of serum creatinine concentration in healthy cats are 0.3 mg/dL.^25^ However, a sustained increase in serum creatinine concentration was utilized in this study and serum creatinine concentration changes were progressive in many cats, despite weight loss, making it likely that there was a definite loss in kidney function over the course of the study. Although BCS was available for most cats in this study, changes in BCS were minimal compared changes in bodyweight, suggesting that BCS might not have been accurately recorded. Use of muscle condition scoring in addition to accurate reporting of BCS is recommended to assist in interpretation of changes in serum creatinine concentration from baseline in future studies.

## CONFLICT OF INTEREST DECLARATION

Robert Rebhun serves as Associate Editor for the Journal of Veterinary Internal Medicine. He was not involved in review of this manuscript.

## OFF‐LABEL ANTIMICROBIAL DECLARATION

Authors declare no off‐label use of antimicrobials.

## INSTITUTIONAL ANIMAL CARE AND USE COMMITTEE (IACUC) OR OTHER APPROVAL DECLARATION

Authors declare no IACUC or other approval was needed.

## HUMAN ETHICS APPROVAL DECLARATION

Authors declare human ethics approval was not needed for this study.
